# Comparison of Ultrasound-Guided Infraclavicular Brachial Plexus Block Sensorial Duration in Diabetic and Non-diabetic Patients: A Prospective Observational Study

**DOI:** 10.5152/TJAR.2022.21402

**Published:** 2022-08-01

**Authors:** Nur Canbolat, Tuğçe Yeniocak, Emine Aysu Salviz, Nukhet Sivrikoz, Kamil Mehmet Tuğrul, Kahraman Öztürk

**Affiliations:** 1 Department of Anaesthesiology, University of Health Sciences, Baltalimani Metin Sabanci Bone and Joint Diseases Education and Research Hospital, İstanbul, Turkey; 2 Department of Anaesthesiology, İstanbul University Faculty of Medicine, İstanbul, Turkey; 3 Department of Hand Surgery, University of Health Sciences, Baltalimani Metin Sabanci Bone and Joint Diseases Education and Research Hospital, İstanbul, Turkey

**Keywords:** Block duration, diabetes mellitus, infraclavicular brachial plexus block, postoperative pain, time-to-first pain

## Abstract

**Objective:**

Diabetic neuropathy is one of the most common complications of diabetes mellitus. Recovery from peripheral nerve blocks in diabetic patients with neuropathy may be delayed because of axonal degeneration and segmental demyelination. The aim of this study is to compare the infraclavicular brachial plexus block durations in patients with and without diabetes mellitus type 2.

**Methods:**

This prospective observational study included 60 patients who were aged 40-80 years, with American Society of Anesthesiologists I-IV physical status and scheduled for elbow, forearm, and/or hand surgery under infraclavicular brachial plexus blocks. All 30 patients in Group DM (patients with diabetes mellitus type 2 diagnosis) and 29/30 patients in Group NODM (patients without diabetes mellitus diagnosis) completed the study successfully. The sensorial block duration was documented as the primary outcome, and the motor block duration, time-to-first pain, numeric rating scale scores at rest/during mobilization, rescue analgesic use, and total consumed doses through the first 2 postoperative days were all documented as the secondary outcomes.

**Results:**

Sensorial block duration in Group DM (505 [315-1020] minutes) was longer than in Group NODM (440 [160-780] minutes) (*P*  = .016). Motor block duration was also longer (488.7 ± 153.8 minutes vs 379.2 ± 118.9 minutes; *P*  = .003), and time-to-first pain was prolonged (625 [360-1200] minutes vs 520 [300-900] minutes; *P*  = .004) in Group DM. The highest NRS scores at the 6th hours, 12th hours, and rescue analgesic consumption through the first 2 postoperative days were lower in Group DM (*P* < .05).

**Conclusion:**

This infraclavicular brachial plexus block study highlights the current literature on diabetic patients with respect to longer block durations, prolonged time-to-first pain, lower pain scores, and less analgesic consumption.

**Main Points**

- Diabetes mellitus is a persistent endocrinopathy with a prevalence of 8.8%, and diabetic neuropathy is one of its most common complications with a high range between 16% and 66%.
- Although peripheral nerve blocks are widely used in diabetic patients worldwide, the knowledge about the association between the nerve blocks and the diabetic neuropathy is very limited.
- In this present study, the sensorial and motor block durations were longer, time-to-first pain was prolonged, and the rescue analgesic requirements were decreased after ultrasound-guided infraclavicular brachial plexus block performances in diabetic patients. In our opinion, it is important to emphasize the possible vulnerability of the diabetic nerves to local anaesthetics and peripheral nerve blocks.

## Introduction

Diabetes mellitus (DM) is a persistent endocrinopathy with a prevalence of 8.8% in 2015 and is predicted to rise to 10.4% in 2040.^1^ Complications of DM increase as the prevalence of DM increases, and diabetic neuropathy (DN) is one of the most common complications with a prevalence of 30%.^2^

In diabetic patients’ anaesthesia management, regional techniques are preferred as opposed to general anaesthesia since surgical stress, hyperglycaemia, and postoperative pain are controlled more successfully.^3^ Despite these advantages, there is still no consensus on the preferred method of anaesthesia for diabetic patients. This is because local anaesthetics (LAs) may deteriorate the condition of nerves with pre-existing neuropathy.^4,5^ Animal and human studies, which mainly contain lower extremity blocks, have demonstrated the association between the diabetic neuropathic nerves and the prolonged duration of block with reduced postoperative analgesics consumption.^5-9^

Infraclavicular (IBPBs) and axillary brachial plexus blocks (ABPBs) are the preferred regional anaesthesia approaches in upper extremity including elbow, forearm, and hand surgeries. Infraclavicular brachial plexus blocks have several advantages compared to ABPBs, such as lower incidence of tourniquet pain, single injection, shorter block performance time, and being better suited for catheter usage.^10-12^ Infraclavicular brachial plexus blocks cause more complications compared to ABPBs; however, these can be decreased by the ultrasound (US) guidance and the experience of the anaesthesiologists.^12^

In this prospective observational study, US-guided IBPB sensorial block durations in diabetic and non-diabetic patients were compared as the primary outcome. Since the literature content about upper extremity nerve block effect in diabetic and non-diabetic patients have been very limited, the null hypothesis testing was planned to be used. The null hypothesis was that sensorial block duration in diabetic patients was comparable to non-diabetic patients. Moreover, the sensorial and motor block onset times, motor block duration, time-to-first pain, highest numeric rating scale (NRS) pain scores, and rescue analgesic consumptions through the first 2 postoperative days were investigated as secondary outcomes.

The present study has been reported according to the Strengthening the Reporting of Observational Studies in Epidemiology guidelines.^13^

## Methods

This prospective, observational study was approved by the İstanbul University Faculty of Medicine Ethics Committee and conducted in Baltalimanı Metin Sabancı Bone and Joint Diseases Education and Research Hospital, Department of Hand Surgery between February 2018 and February 2019. Written informed consent was obtained from each participant after the enlightenment of anaesthetic technique, postoperative analgesia options, and publication of the present study. Inclusion criteria were aged 40-80 years and American Society of Anaesthesiologists (ASA) I-IV patients scheduled to undergo elbow, forearm, and/or hand surgeries under US-guided IBPBs. Exclusion criteria were contraindication to regional anaesthesia, known allergy to LAs, difficulty in understanding instructions and pain scales, cognitive impairment, alcohol and/or drug abuse including opioids, neurologic disorders of the upper extremity, only diet-controlled DM type 2, and also DM type 1 diagnosis.

Taking into consideration a similar study that compared sensory block duration in diabetic and non-diabetic patients after ABPBs,^9^ a pilot study was designed to obtain a 25% difference for the same outcome and 19 patients/group were planned to be enrolled in this study. In order to compensate for follow-up/data losses, 30 patients/group were included in each group. During the study period, after each diabetic patient was included in Group DM, the consecutive non-diabetic patient was included in Group NODM (patients without diabetes mellitus diagnosis) until the required number of patients was reached.

On the day of the surgery, standard monitoring was applied to each of 60 patients in a dedicated block procedure room. All patients were premedicated with 2 mg midazolam and 50 µg IV fentanyl before the IBPB and given O_2_ 2 L h^−1^ via facemask. The patient demographics (sex, age, body mass index [BMI]) and ASA physical statuses were recorded. Fasting blood glucose and haemoglobin A1c (HbA1c) levels, time since diagnosis, use of oral anti-diabetic and/or insulin therapies, and also the presence of diabetic peripheral neuropathy (diagnosed by endocrinologists after the performance of pin-prick sensation, strength/muscle atrophy, deep tendon reflex, skin assessment, and Tinel tests) of the Group DM patients were collected.

### Ultrasound- G uided Infraclavicular Brachial Plexus Blocks

Infraclavicular brachial plexus blocks (coracoid approach) were performed by administering a LA mixture of 15 mL lidocaine 2% and 15 mL bupivacaine 0.5% at the 6 o’clock position to the subclavian/axillary artery under US guidance. In addition to US, nerve stimulator (NS) was always used to avoid inadvertent neural injury. All IBPBs were performed by 2 independent anaesthesiologists, who have the same substantial expertise and were blinded to the DM diagnosis of the patients.

Block performance duration was defined as the time interval between the first insertion of the needle and its final removal and was recorded. In addition, incidences of paraesthesia that occurred due to needle-nerve contact and vascular puncture were noted. The regions of the radial, median, ulnar, and musculocutaneous nerves were assessed every 5 minutes up to 30 minutes or until the block onset again by other investigators blinded to the study and were documented. Sensorial block was evaluated by using a cold test on the dermatomes of the median, ulnar, radial, and musculocutaneous nerves. Sensorial block was graded on a 3-point scale (0: normal sensation, 1: analgesia [patient can feel touch but not cold], 2: anaesthesia [patient cannot even feel the touch]). The sensorial block onset time was defined as the time to reach an absent sensation in all 4 nerve distributions after the completion of LA injection (2 points in all 4 sensation areas or 2 points in 3 and 1 point on the remaining 1 sensation area: ≥7/8). Motor block was graded on a 4-point scale (0: normal strength; 1: partial motor block, able to flex the elbow and move the fingers but unable to raise the extended arm; 2: almost complete motor block, unable to flex the elbow but able to move the fingers; and 3: complete motor block). The motor block onset time was described as the time to reach complete motor block (3 points in at least 3 different motor functions or 3 points in 2 motor functions and 2 points in the remaining 2 other motor functions: ≥9/12) after the completion of LA deposition.

Block failure was defined as failure to achieve surgical anaesthesia at the 30th minute after the IBPB or pain at the operative site during surgery. The patient, who experienced a failed block, underwent the surgery under general anaesthesia and was excluded from the study. The surgery types and durations of the patients with successful IBPBs and uneventful surgeries were recorded for the study.

All patients in both groups were given 2×1 g acetaminophen IV daily as a standard postoperative analgesic treatment and were asked to rate their pain at 0th min, 1st, 2nd, 6th, 12th, 24th, and 48th hours using NRS pain scores (ordinal data, 0-10 scale: 0: no pain; 10: the worst pain imaginable). If NRS was ≥4, 75 mg diclofenac sodium IM was applied as a rescue analgesic (maximum dose: 2×1). If NRS was again ≥4 after an hour or before the second diclofenac sodium dosing time reached, additional 100 mg IV tramadol (maximum dose 2× 100 mg) was administered as the second rescue analgesic. The number of patients who used rescue analgesics and the total rescue analgesic consumption during the first postoperative 2 days were both noted.

The sensorial and motor block durations were identified as the time intervals between a successful block and the complete restoration of all senses controlled by the radial, ulnar, median, and musculocutaneous nerves and the complete recovery of motor functions of the same nerves, respectively. Time-to-first pain was determined at the first postoperative time point that NRS was ≥4. In order to obtain the exact duration data, the patients and their family members were educated preoperatively for informing the investigators about the first move, sensorial feeling, and pain. In addition, frequent visits and physical examinations were performed not only at NRS score time points (0th, 1st, 2nd, 6th,12th, 24th, and 48th hours) but also in between. Postoperative data such as sensorial block duration (primary outcome), motor block duration, time-to-first pain, postoperative highest NRS pain scores at rest/during mobilisation, and rescue analgesic consumptions through the first 2 postoperative days (secondary outcomes) were all documented by anaesthesiologists who were blinded to the patients’ diabetes status.

### Statistical A nalysis

A study by Salviz et al^9^ compared sensory block duration in diabetic and non-diabetic patients who underwent upper extremity surgeries under ABPBs. Their sensorial block duration was 779 ± 197 minutes in diabetic patients. In order to detect a clinically significant 25% difference, with an α error of 0.05 and a β error of 0.20, it was calculated that at least 19 patients/group would be required. Thirty patients per group were planned to be included to compensate for the possible follow-up/data losses.

Continuous data are presented as mean ± standard deviation, as appropriate. Student *t*-test was used for parametric data such as age, BMI, and motor block duration. Mann–Whitney *U* test was used for non-parametric data such as block performance duration, surgery duration, sensorial block duration, time-to-first pain, NRS at rest/during mobilisation, and rescue analgesic consumption. One of Pearson chi-square (χ^2^) or Fischer’s exact tests was used for categorical variables such as sex, ASA physical status, paraesthesia and vascular puncture incidence during the block, surgery types, sensorial and motor block onset times at given time points, and number of patients used rescue analgesics. After comparing diabetic and non-diabetic patients, a multivariable regression was performed using sensorial block duration (primary outcome) as the dependent variable and the presence of DM, ASA III-IV status, sex, and BMI characteristics as independent variables. Moreover, the correlation between the time since the diagnosis of DM and sensorial block duration and also HbA1c levels and sensorial block duration was calculated. *P*  < .05 was considered statistically significant.

## Results

**Figure 1.**
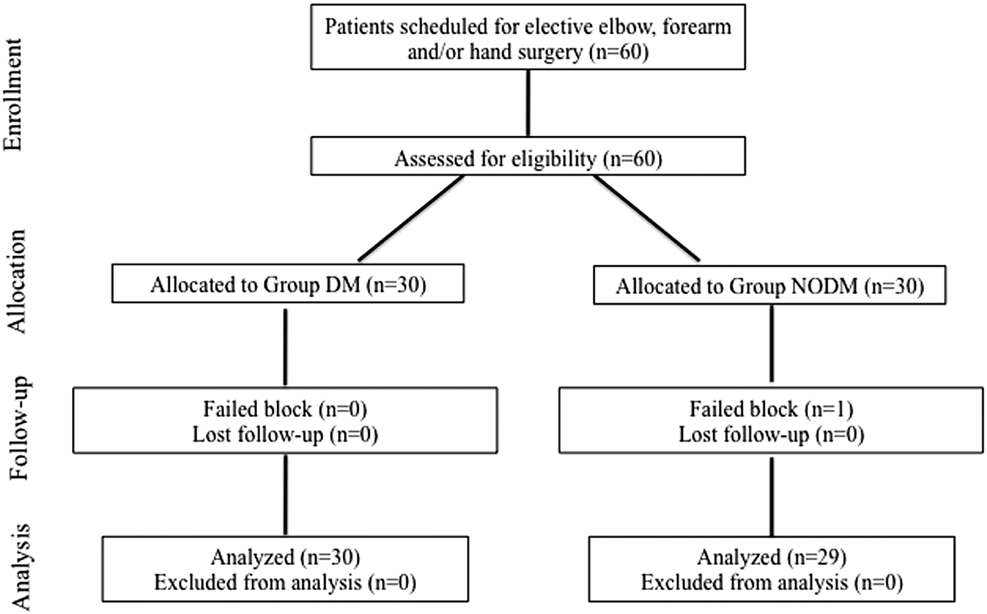
Strengthening the Reporting of Observational Studies in Epidemiology diagram of Groups DM and NODM, who received infraclavicular brachial plexus block. Group DM, patients with diabetes mellitus; Group NODM, patients without diabetes mellitus.

Sixty patients, who were scheduled for elbow, forearm, and/or hand surgery, were eligible and enrolled to 1 of the 2 groups according to their DM diagnosis. Only 1 patient was excluded from Group NODM because of a failed block. In total, 59 patients completed the study: 30 in Group DM and 29 in Group NODM (Figure 1).

In terms of age and gender, patients’ demographic data were similar in both groups (*P*  > .05). However, as shown in Table 1, Group DM patients were more obese and had higher ASA physical status. Table 2 demonstrates features of diabetic patients.

[Table t2-tjar-50-4-267]

**Figure 2.**
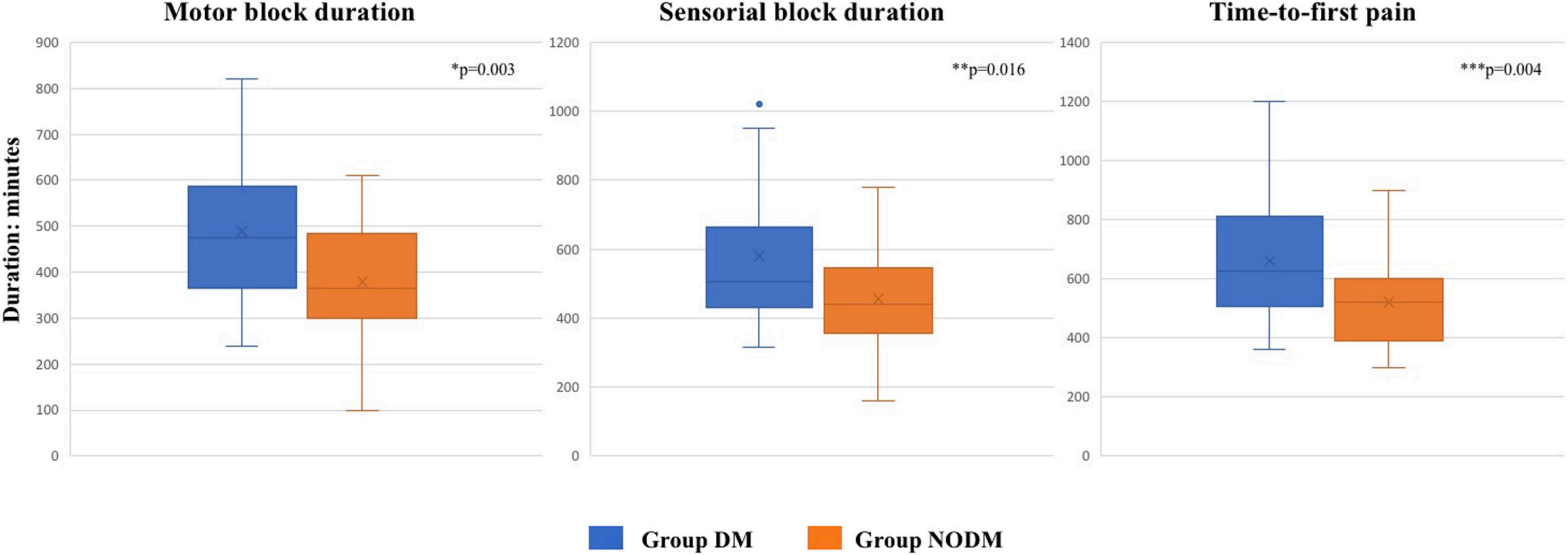
Prolonged motor block duration*, sensorial block duration**, and time-to-first pain*** of Group DM compared to Group NODM are shown. Group DM, patients with diabetes mellitus; Group NODM, patients without diabetes mellitus.

Sensorial block duration in Group DM (505 [315-1020] minutes) was significantly longer than in Group NODM (440 [160-780] minutes) (*P*  = .016) (Figure 2). A multivariate regression was performed in this study using postoperative sensorial block duration as the dependent variable and the presence of DM, ASA III-IV status, sex, and BMI as independent variables (significant variables were detected). Only the presence of DM was found to be associated with prolonged sensorial block duration (*P*  = .01; coefficient of determination = 0.120; multiple correlation coefficient = 0.346). Bivariate correlation was also performed; however, no correlation was shown between sensorial block duration and time since DM diagnosis (*P*  = .716), and also sensorial block duration and HbA1c levels (*P*  = .796).

The surgery types of those patients who underwent surgeries such as open carpal tunnel release, distal radius fracture, olecranon fracture, scaphotrapeziotrapezoid and carpometacarpal arthritis, tenosynovitis (Dequervain's release), soft tissue tumor excision, debridement, tendon repair surgery, Dupuytren contracture, mallet finger repair, nerve injury repair, and hand and finger(s) injury/amputation/replantation surgeries were similar in both groups (*P*  = .93).

Clinical and also block characteristics of patients are demonstrated in Table 3. The proportion of patients defined as ≥7/8 sensorial block and ≥9/12 motor block at every 5 minutes until 30 minutes or until the onset of block was similar in Groups DM and NODM. Motor block duration was longer in Group DM (488.7 ± 153.8 minutes) compared with Group NODM (379.2 ± 118.9 minutes) (*P*  = .003). Time-to-first pain was prolonged in Group DM compared to Group NODM (625 [360-1200] minutes and 520 [300-900] minutes, respectively) (*P*  = .004) (Figure 2).

The highest NRS scores at 6th and 12th hours were lower in Group DM (*P*  < .05) (Table 4). The number of patients requested diclofenac sodium on postoperative first day (*P*  = .045) and second day (*P*  = .03), and tramadol as the second rescue analgesic on postoperative first day (*P*  = .033) were all significantly fewer in Group DM. Similarly, patients in Group DM consumed less diclofenac sodium on postoperative days 1 (*P*  = .026) and 2 (*P*  = .003), and tramadol on only day 1 (*P*  = .02). The highest NRS score at rest at 24th hour was also lower, but not significant (*P*  = .051). No complication was observed in any of the patients.

## Discussion

In the current study, a longer sensorial block duration in diabetic patients was obtained. Motor block duration and time-to-first pain were both also prolonged in diabetic patients compared to non-diabetic patients. Moreover, the highest NRS scores at the 6th and 12th hours were lower, and the rescue analgesic requirements were decreased through the postoperative first 2 days.

Peripheral neuropathy is a microvascular complication of DM with a high range between 16% and 66% in different studies.^14^ Asymptomatic peripheral neuropathy is also very common in diabetic patients.^15^ In a recent 2018 study, Yang et al^16^ examined 127 type 2 diabetic patients using electrophysiological tests for median and ulnar nerves and found that only 9 (7.1%) of the patients have not had any types of neuropathy. Gündüz et al^17^ also examined the presence of ulnar neuropathy at the elbow level in diabetic patients and only 1/36 patients declared symptoms suggesting neuropathy. Characteristics of sciatic nerve blocks had been compared in non-diabetic and diabetic patients with and without DN in various studies.^8,18^ The time-to-first analgesic request was longer in diabetic patients compared to non-diabetic patients. In this study, 7/30 (23.3%) diabetic patients had clinical DN according to the endocrinologists’ examinations of pin-prick sensation, strength/muscle atrophy, deep tendon reflex, skin assessment, and Tinel tests. Additional test for DN diagnosis was not performed; however, we know from previous studies that asymptomatic DN is also very common, and the patients might have inchoate nerve injury that could be undetectable.^15^ There are 2 mechanisms attributed to the longer nerve block durations in DN: (i) DM reduces the activity of potassium and sodium channels in the nerve fibres, influencing the threshold and the conduction velocity in these neurons,^4,19^ and (ii) LA washout time in neurons is prolonged.^20^ Microvascular dysfunction reduces the absorption of LAs in neuropathic nerves and increases the block duration.^7^ These explanations may describe our longer sensorial and motor block durations and are also associated with prolonged time-to-first pain findings in Group DM patients.

Nevertheless, apart from the presence of DM, there was no other correlation for sensorial block duration. Ziegler et al^21^ showed the DM duration as a major risk factor for distal sensorimotor polyneuropathy, and Salviz et al^9^ reported the relationship between DM duration and sensory block duration after ABPBs. On the other hand, Sertoz et al^22^ found that patients with worse DM regulation (higher HbA1c levels) had longer block duration. The uncorrelated results of the current study may be explained by well-controlled short- (preoperative fasting) and long-term (HbA1c) blood glucose levels, ≤10 years of DM diagnosis time, and low peripheral neuropathy diagnosis incidence (23.3%).

In the present study, patients in both groups had similar NRS scores at rest and also during mobilisation through the postoperative first 6 hours, which can easily be attributed to the successful IBPBs. Subsequently, lower NRS scores, especially at about the 6th and 12th hours and sometimes at the 24th hour, were observed in patients with DM due to longer block and analgesia durations. At these time points, NRS scores themselves and also the NRS score differences between groups were at their highest levels because of the distinctive block effect withdrawal times. In non-diabetic patients, mobilisation of any part of the upper extremity caused more pain at the same time points and they consumed larger doses of rescue analgesics. All these outcomes of this present study supported the previous upper extremity study performed by Salviz et al.^9^

Clinical studies have conflicting results for the onset time of peripheral nerve blocks. Sertoz et al^22^ demonstrated delayed sciatic nerve block onset time in diabetic patients, especially who were with higher HbA1c levels, whereas others did not find any difference.^7,9^ Chronic hyperglycaemia induces inflammation, oxidative stress, and mitochondrial dysfunction in nerve fibres. Axonal degeneration and segmental demyelination are the pathologic characteristics of neuropathic nerves. Damaged nerves are less sensitive to stimulation^4,23^ but expected to be more sensitive to LAs.^24^ However, in this study, no difference was found between the onset times of nerve blocks. This result might be obtained due to the well-controlled blood glucose levels of the participants (fasting blood glucose level <200 g dL^−1^ and HbA1c level <6.5%).

In a retrospective study, supraclavicular nerve block success rate was higher in diabetic patients (96% in patients with DM vs 87% in patients without DM).^25^ In our study, only 1 patient from Group NODM was excluded because of a failed block. The high success rate across study groups was probably due to US guidance that is used in addition to NS guidance. Ultrasound-guided IBPBs have already been shown to have a shorter procedure time and a higher success rate.^26^

The rate of obese patients was slightly higher in the DM group. Since the doses of LAs used in peripheral nerve blocks should be adjusted according to the ideal body weight, the standard dose and volume we routinely used were within the range of successful and also safe treatment limits for our study patients. Nevertheless, the possibility of nerve deterioration in diabetic patients with known or unknown pre-existing pathologies was always kept in mind.^4,5^ After searching the literature and obtaining the longer/prolonged block effects in diabetic patients during this investigation once more, we have switched our daily routine practice to use lower doses for diabetic patients’ peripheral nerve blocks.

There have been some limitations in the current research. First, the study included different types of minor and major orthopaedic surgeries. However, compared to the previous upper extremity study,^9^ this study included a bigger number of major and painful surgeries, which may contribute to the understanding of the blocks’ effects. Second, supplemental DN-related monofilament tests were not performed by anaesthesiologists and the study relies on the endocrinologists’ results. Nevertheless, as asymptomatic and undiagnosed peripheral neuropathy is very common,^15^ we could not make sure whether any new information would have been added to the patients’ neuropathy incidence or to the results.

## Conclusion

Consequently, prolonged sensorial and motor block durations and associated increased time-to-first pain were observed after application of US-guided IBPB on diabetic patients. As the diabetic nerves are thought to be more sensitive to LAs, further prospective studies with large number of patients should be performed to investigate the effect of different US-guided peripheral nerve blocks, the effect of various LAs with lower doses in order to modify the routine practices, if necessary, and to avoid the nerve injuries.

## Figures and Tables

**Table 1.** Patient Demographics

	**Group DM (*n* = 30)**	**Group NODM (*n* = 29)**	***P***
**Sex (F/M)**	12/18	15/14	.366
**Age (years)**	54.93 ± 9.17	51.66 ± 7.07	.130
**BMI (kg m−2 )**	29.24 ± 4.42	27 ± 3.92	.045
**ASA physical status, **n** (%)**			
I	0/30 (0)	10/29 (34.5)	.001
II	28/30 (93.3)	19/29 (65.5)	
III	2/30 (6.7)	0/29 (0)

Data are presented as mean ± standard deviation (Student-t Test) and n (%) (Pearson Chi-Square [χ^2^] or Fisher’s Exact Test [when n ≤ 5]). Group DM, patients with diabetes mellitus group; NODM, patients without diabetes mellitus; BMI, body mass index; ASA, American Society of Anesthesiologists.

**Table 2. t2-tjar-50-4-267:** Features of Patients with Diabetes Mellitus

	**Group DM (*n* = 30)**
Fasting blood glucose levels (g dL−1)	129.5 (88-196)
HbA1c levels (%)	6.02 ± 0.018
Time since diagnosis (years)	4.5 (1-20)
Patients with oral anti-diabetic therapy (n [%])	30/30 (100%)
Patients with insulin therapy (n [%])	10/30 (33.3%)
Patients with peripheral neuropathy (n [%])	7/30 (23.3%)

Data are presented as median (min-max), mean ± standard deviation and n (%). Group DM, patients with diabetes mellitus.

**Table 3.** Clinical and Block Characteristics of Patients Observed in Both Groups

	**Group DM (*n* = 30)**	**Group NODM (*n* = 29)**	***P***
Block performance duration (min)	5 (3-12)	7 (3-10)	.119
Paraesthesia incidence during the block, *n* (%)	0/30 (0)	0/29 (0)	
Vascular puncture incidence during the block, *n* (%)	0/30 (0)	1/29 (3.4%)	.492
Surgery duration (min)	60 (20-145)	88 (25-134)	.271
**Sensory block onset time, **n**(%)**			
0th minute	0/30 (0)	0/29 (0)	
5th minute	6/30 (20)	6/29 (20.7)	.948
10th minute	17/30 (56.7)	16/29 (55.2)	.908
15th minute	20/30 (66.7)	18/29 (62.1)	.712
20th minute	24/30 (80)	24/29 (82.8)	.786
25th minute	29/30 (96.7)	27/29 (93.1)	.612
30th minute	30/30 (100)	29/29 (100)	
**Motor block onset time, **n**(%)**			
0th minute	0/30 (0)	0/29 (0)	
5th minute	4/30 (13.3)	3/29 (10.3)	1
10th minute	12/30 (40)	9/29 (31)	.472
15th minute	16/30 (53.3)	15/29 (51.7)	.902
20th minute	20/30 (66.7)	18/29 (62.1)	.902
25th minute	25/30 (83.3)	24/29 (82.8)	1
30th minute	30/30 (100)	29/29 (100)	

Data are presented as median (min-max) (Mann–Whitney *U* test) and n (%) (Pearson Chi-Square [*χ ^2^*] or Fisher’s Exact Test [when n ≤ 5]). Group DM, patients with diabetes mellitus; Group NODM, patients without diabetes mellitus.

**Table 4.** The highest NRS Scores

	**Group DM (*n* = 30)**	**Group NODM (*n* = 29)**	***P***
**NRS at rest**			
0th minute	0 (0-2)	0 (0-2)	.981
1st hour	0 (0-2)	0 (0-2)	.604
2nd hour	0 (0-2)	0 (0-2)	.471
6th hour	0 (0-4)	2 (0-6)	.035
12th hour	2 (0-4)	3 (0-5)	.043
24th hour	1 (0-4)	2 (0-6)	.051
48th hour	0 (0-2)	1 (0-4)	.064
**NRS during mobilisation**			
0th minute	0 (0-2)	0 (0-2)	.229
1st hour	0 (0-2)	0 (0-2)	.193
2nd hour	0 (0-3)	0 (0-3)	.487
6th hour	1 (0-6)	2 (0-7)	.026
12th hour	2 (0-7)	3 (1-8)	.007
24th hour	1 (0-5)	2 (0-6)	.090
48th hour	0 (0-3)	1 (0-4)	.140

NRS, numeric rating scale (0: no pain, 10: worst pain imaginable). Data are presented as median (min-max) (Mann–Whitney *U* test) and n (%) (Pearson Chi-Square [χ^2^] or Fisher’s Exact Test [when n ≤ 5]). Group DM: Patients with Dabetes Mellitus, Group NODM: Patients Without Diabetes Mellitus.
